# Patient and clinician-reported experiences of using electronic patient reported outcome measures (ePROMs) as part of routine cancer care

**DOI:** 10.1186/s41687-023-00544-4

**Published:** 2023-05-04

**Authors:** Amelia Payne, Ashley Horne, Neil Bayman, Fiona Blackhall, Layla Bostock, Clara Chan, Joanna Coote, Marie Eaton, Jacqueline Fenemore, Fabio Gomes, Emma Halkyard, Margaret Harris, Colin Lindsay, Delyth McEntee, Hilary Neal, Laura Pemberton, Hamid Sheikh, David Woolf, James Price, Janelle Yorke, Corinne Faivre-Finn

**Affiliations:** 1 University of Manchester, Manchester, UK; 2 Christie NHS Foundation Trust, Manchester, UK

**Keywords:** Patient reported outcomes, Electronic questionnaires, Cancer, Patient experience, ePROMs, PROMs

## Abstract

**Background:**

Cancer and its treatment can have significant impacts on health status, quality of life and functioning of patients. Direct information from patients regarding these aspects can be collected via electronic platforms in the form of electronic Patient Reported Outcome Measures (ePROMs). Research has shown that the use of ePROMS in cancer care leads to improved communication, better symptom control, prolonged survival and a reduction in hospital admissions and emergency department attendance. Acceptability and feasibility of routine ePROM collection has been reported by both patients and clinicians but to date their use has predominantly been limited to clinical trials. MyChristie-MyHealth is an initiative from a UK comprehensive cancer centre The Christie NHS Foundation Trust which incorporates the regular collection of ePROMs into routine cancer care. This study, carried out as part of a service evaluation, explores patient and clinician experiences of using the MyChristie-MyHealth ePROMs service.

**Results:**

100 patients with lung and head and neck cancers completed a Patient Reported Experience questionnaire. All patients reported that MyChristie-MyHealth was easy to understand and, almost all found it timely to complete and easy to follow. Most patients (82%) reported it improved their communication with their oncology team and helped them to feel more involved with their care (88%). A large proportion of clinicians (8/11) felt ePROMs helped communication with their patients and over half (6/10) felt they led to consultations being more patient focused. Clinicians also felt that the use of ePROMs resulted in patients being more engaged in consultations (7/11) and their cancer care in general (5/11). Five clinicians reported that the use of ePROMs altered their clinical decision making.

**Conclusions:**

Regular ePROMs collection as part of routine cancer care is acceptable to both patients and clinicians. Both patients and clinicians feel their use improved communication and increased the feeling of patient involvement with their care. Further work is needed to explore the experiences of patients that did not complete the ePROMs as part of the initiative and to continue to optimize the service for both patients and clinicians.

**Supplementary Information:**

The online version contains supplementary material available at 10.1186/s41687-023-00544-4.

## Background

There are over two and a half million people in the UK currently living with cancer and this number is set to increase to 4 million by 2030 [1]. The symptom burden for these patients can be high [2, 3] and even mild side-effects can impact quality of life (QoL) and lead to cessation of treatment, especially with prolonged treatment regimes [4]. The effective management of cancer symptoms or treatment-related side-effects is integral to maintaining a good QoL in patients living with cancer.

Patient Reported Outcome Measures (PROMs) are used to gather information about health status, QoL and functioning directly from patients, without any interpretation from a member of clinical staff [5]. PROMs allow patients to report on symptom severity as well as the impact of these symptoms on QoL, functioning and overall well-being. The benefits of integrating remotely-reported PROMs using electronic platforms (ePROMs) within the clinical pathways are well documented [6–11]. Randomised controlled trials have demonstrated that the use of ePROMs lead to improvements in the doctor/patient relationship as a result of enhanced communication and clinical efficiency, better symptom control, reduced emergency department attendance, reduced hospitalisation and improved survival. ePROMs have also been shown to lead to earlier detection [12] and management [13, 14] of symptoms as well as earlier detection of tumour recurrence [15]. Furthermore, automated feedback to patients on completing ePROMs can identify milder symptoms which do not necessarily need clinician involvement and can be managed at home [4]. On the whole, the routine collection of ePROMs can enable a more holistic and patient-centred approach to clinical care [16–18]. This high level evidence has been invaluable in this arena for informing shared decision-making as well as economic and regulatory analyses [6–8].

To date, the implementation of ePROMs in oncology has mostly occurred in the context of clinical trials while their integration into routine cancer care is still to be established. Patients and clinicians report high satisfaction and acceptability when ePROMs are used as part of routine cancer care [19]. However, a number of patient, clinician and logistical barriers to ePROMs integration in this setting should be taken into consideration in order to make the routine implementation of ePROMs a reality [19–27].

‘MyChristie-MyHealth’ was launched in January 2019, integrating ePROM questionnaires routinely into patient care pathways [28]. As part of the evaluation of the ePROMs service, we aimed to assess the acceptability and feasibility of regular ePROMs collection in routine cancer care and explore patient and clinician experiences of the service.

## Methods

### Study design

This was a single-centre, questionnaire-based study which formed part of a service evaluation of the MyChristie-MyHealth initiative. The study focused on patients with lung cancer and head and neck cancer, the two main disease groups in which this service was initially introduced.

The aim was to demonstrate the feasibility of ePROMs collection in routine cancer care and to explore patient and clinician experiences of the service. This service evaluation was reviewed and approved by the Christie NHS Foundation Trust Governance Panel.

### MyChristie-MyHealth ePROMs service

**Fig. 1:**
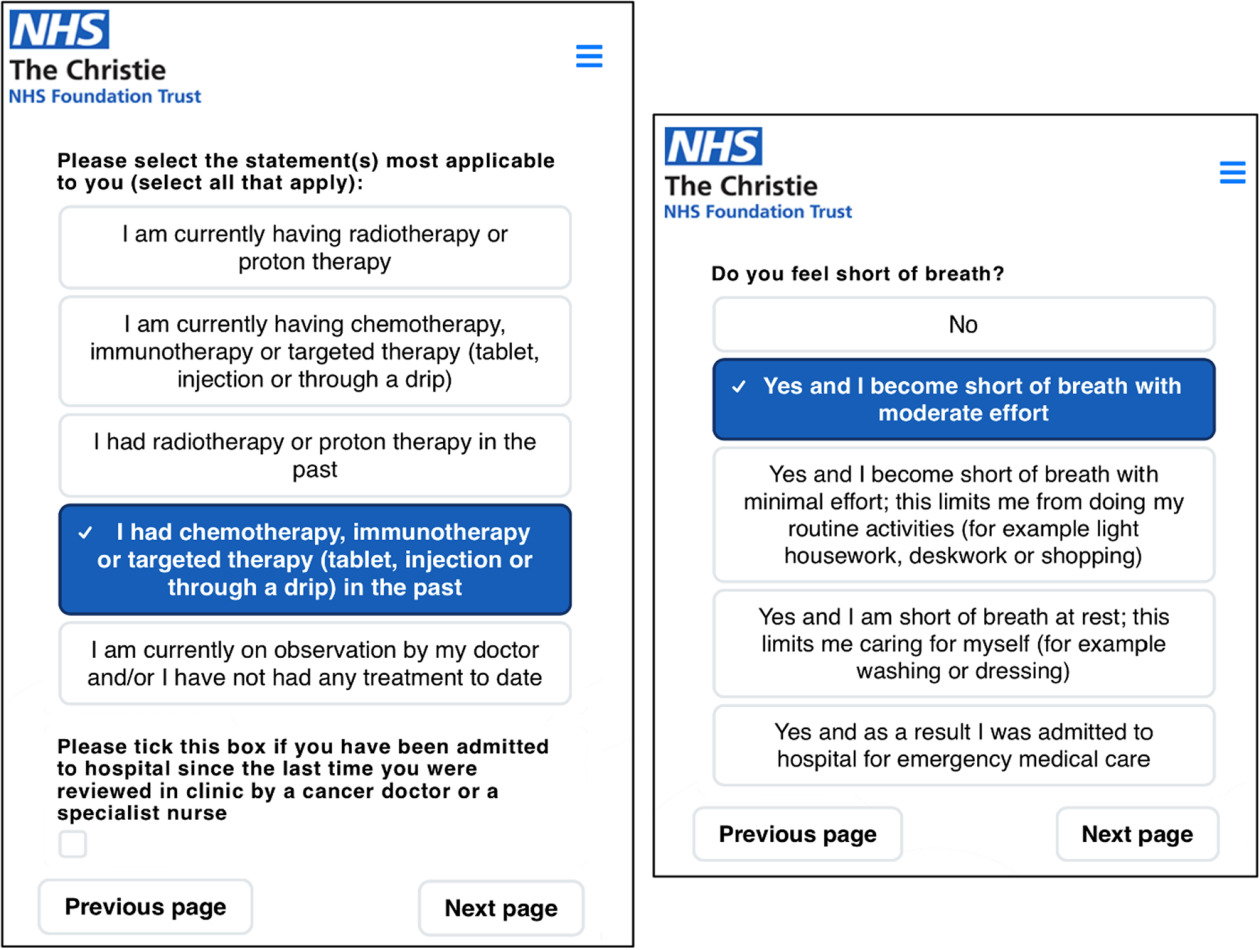
Example of ePROM questions

**Fig. 2:**
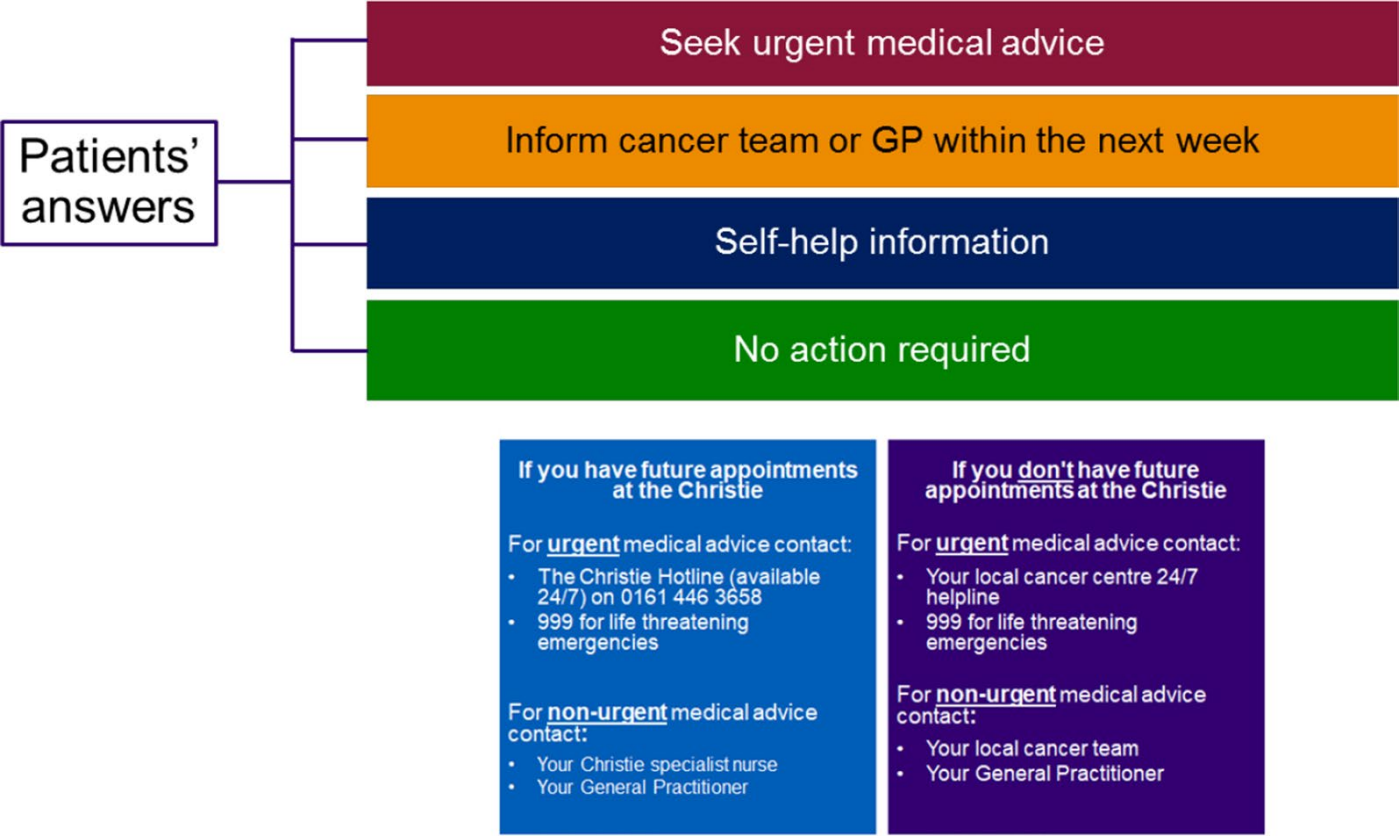
Schematic of patient alert messages

Patients with an outpatient consultation automatically receive a text message or email containing a personalised link to access the MyChristie-MyHealth platform the day before their first clinic appointment or three days prior to a scheduled follow-up appointment. Patients then log onto the ePROM platform using their personal details (surname, date of birth and postcode) to complete the questionnaire. Patients were able to seek assistance to complete ePROMs via a proxy (e.g. family member) or a member of the Christie ePROM team. This help was solely of a technical nature, such as logging on to the MyChristie-MyHealth platform, and all ePROMS responses were entirely the patient’s own (Figs. 1 and 2).

The ePROMs questionnaire consist of symptom items written in lay language, adapted from the Common Terminology Criteria for Adverse Events (v5.0) [29] and quality of life items (using the EuroQol EQ-5D-5L quality of life (QoL) tool [30]. Symptom items were chosen by the relevant clinical teams. Patients and specialist nurses were involved with the development of the MyChristie-MyHealth ePROM questionnaire. The type and number of symptom items is dependent on prior treatment received, e.g., systemic anticancer therapy or radiotherapy. Examples of the ePROMs questionnaires used in the lung and head and neck patient groups are provided in Additional file 1: Appendix 1 and Additional file 2: Appendix 2.

Following completion of an ePROMs questionnaire, patients are presented with colour-coded advice dependent on symptom severity. Patients without any symptoms receive a message reassuring them that no action is required (green). Those with mild symptoms receive an alert with a link to the Macmillan website [31] that includes self-care advice (blue). Moderate symptoms elicit advice to seek medical attention from their oncology team or their General Practitioner within a week (orange). Finally, those with severe symptoms receive an alert advising them to seek urgent medical advice within 24 h (red). Each advice alert was accompanied by the hospital’s 24/7 hotline contact details.

For the purposes of this report, data regarding demographics, disease stage, performance status and comorbidity burden (Adult Comorbidity Evaluation (ACE) score) were collected prospectively at the time of consultations by the clinical teams. Missing data was collected by the first author from the electronic patient record.

At the time when this study was conducted, the results of the ePROMs questionnaires were not integrated into the institution’s electronic patient record. In order to view the completed ePROMS questionnaires clinicians logged into a separate electronic platform, which was provided by a digital health company (DrDoctor®), and they were encouraged to do so prior to each clinical encounter. Clinicians were reminded by members of the ePROM team to log onto the platform and review responses, at the start of each clinic.

### Patient experience

#### Participants

Patients who attended lung cancer or head and neck cancer clinics between May 2019 and June 2019 and had completed at least one ePROMs questionnaire were invited to complete a Patient Reported Experience Measure (PREM) questionnaire. All consecutive patients who attended these clinics were approached to complete the questionnaire.

Participants were excluded if they had not completed an ePROMs questionnaire prior to the assessment period, if they had completed the questionnaire on the day of PREM collection with the help of a member of the ePROMs team, or if they had completed the questionnaire with the assistance of a proxy who was not present at the time of PREM collection.

#### Questionnaire development and content

The PREM questionnaire was developed in collaboration with the Christie ePROMs Steering Group. Questions were chosen by the ePROMs steering group and formed into draft questionnaires. Clinicians, clinical nurse specialists and patient representatives were asked to review the questionnaires to ensure they were relevant and understandable, and provided modifications where needed. The final questionnaires were reviewed and approved by the ePROMs steering group prior to roll-out.

The questionnaire consisted of six questions exploring the usability of the ePROMs questionnaire, the timing of the text messages and the impact on clinical care. These questions were answered using a 4 point Likert scale (from 1 ‘strongly agree’ to 4 ‘strongly disagree’). A neutral option was omitted as research has suggested that 10–20% of those who answer with a neutral option tend to have a preference either favourably or unfavourably [32]. After discussion within the ePROM steering group the neutral option was omitted for this reason.

**Fig. 3:**
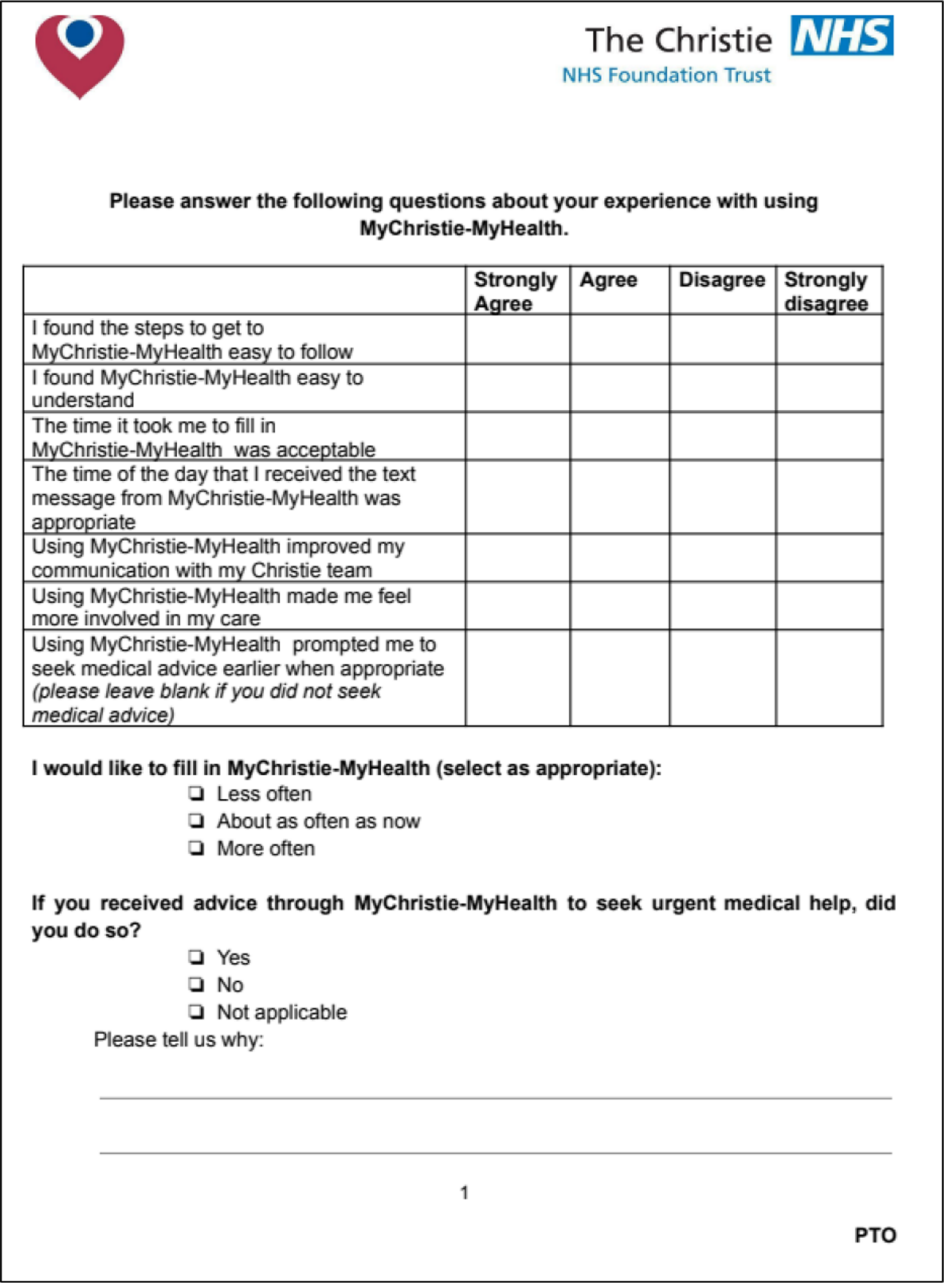

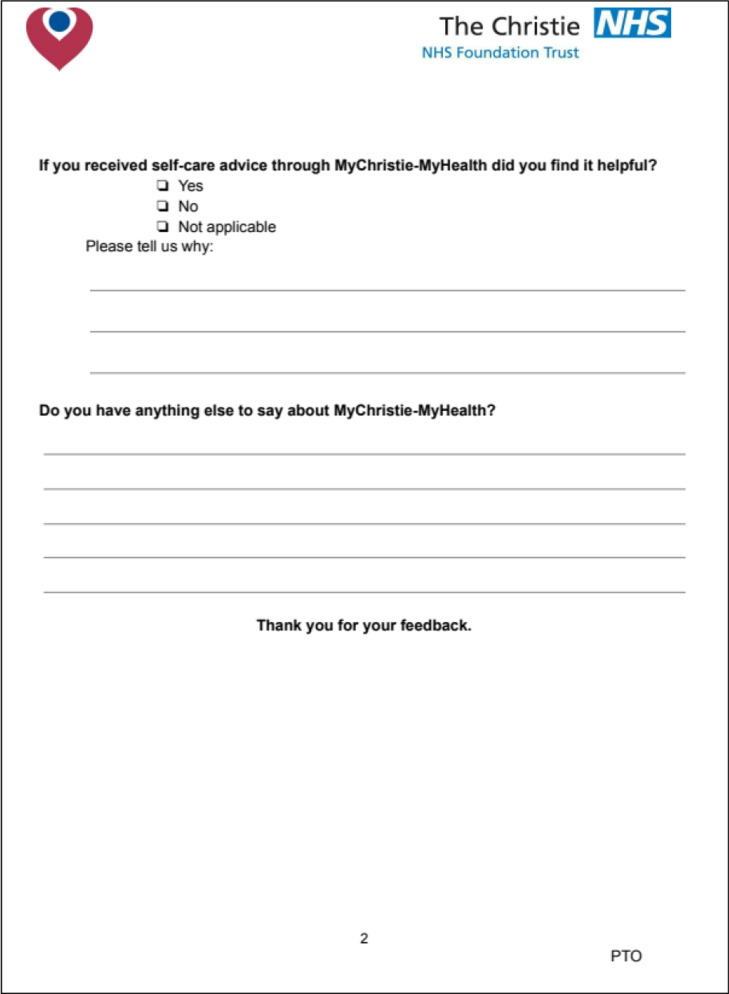
Patient experience measure (PREM) questionnaire

A further two dichotomous (‘yes/no’) questions with free text boxes were added to gain information regarding the advice messages and the frequency of questionnaire administration. A final free text box was added at the end for further comments about the MyChristie-MyHealth service (Fig. 3).

Both paper and electronic versions were available to allow as many patients as possible to participate. All paper versions were anonymised, entered onto the ePROMs platform and subsequently disposed of securely.

### Clinician experience

#### Participants

All clinicians involved in lung and head and neck cancer clinics between May 2019 and July 2019, were invited to complete a clinician experience questionnaire. Participants were approached in person and via email.

#### Questionnaire development and content

The clinician questionnaire was also developed with input from the Christie ePROMs steering committee Potential questions to be included were discussed with steering group, these were then constructed into a questionnaire that was reviewed and adapted by the steering group. After final review and approval this was uploaded and distributed using an online platform.

**Fig. 4:**
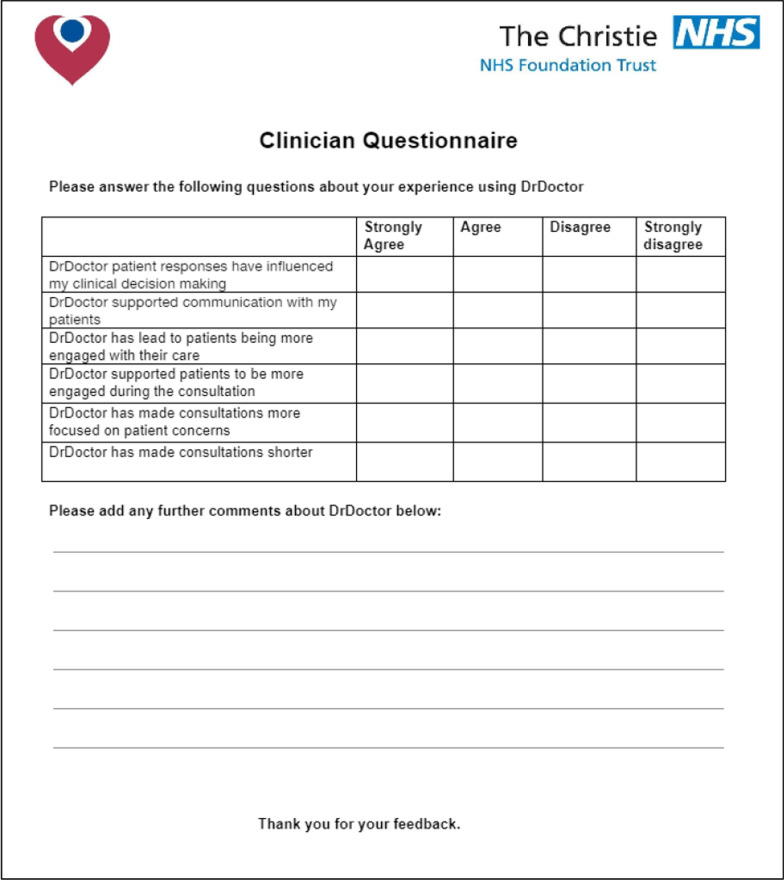
Clinician experience questionnaire

It included six questions using a 4 point Likert scale, as outlined above. These questions explored the impact of the service on clinical decision making, communication with patients, duration of consultations and patient engagement in their consultation and their clinical care as a whole. The clinician questionnaire is shown in Fig. 4 below.

## Results

### Patient experience

#### Study population

Between May and July 2019 107 patients were approached to complete a PREM. Of these 100 PREMs were returned completed. Two patients declined due to anxiety around upcoming appointment, 1 completed the ePROM with a proxy who was not present at the time of PREM collection and four were returned with incomplete data that was insufficient of analysis. The median patient age was 67 years (range 30–80 years) and 50% were female. 78 patients had lung cancer and 22 had a head and neck cancer (Table 1). Most patients had an ECOG performance status of 0–1 (86%) and the remainder (14%) had a performance status of 2. Seventy-five percent of patients had an ACE-27 score of 0–1. Almost half (49%) of patients had non-metastatic disease, 42% had metastatic or extensive stage disease and for the remainder, the extent of disease was not documented (Table 1).

**Table 1:** Patient demographic data

Patient demographics			
Characteristic	All N = 100 n*(%)*	Lung N = 78 n*(%)*	Head and neck N = 22 n*(%)*
**Age (years)**			
Median (range)	67 (30–80)	68.5 (40–80)	63.5 (30–74)
**Gender**			
Male	50 *(50.0)*	36 *(46.2)*	14 *(63.6)*
Female	50 *(50.0)*	42 *(53.8)*	8 *(36.4)*
**Stage**			
I	3 *(3.0)*	2 *(2.6)*	1 *(4.5)*
II	6 *(6.0)*	2 *(2.5)*	4 *(18.2)*
III	38 *(38.0)*	32 *(41.1)*	6 *(27.3)*
IV	41 *(41.0)*	36 *(46.2)*	5 *(22.7)*
Limited*	2 *(2.0)*	2 *(2.6)*	-
Extensive*	2 *(2.0)*	2 *(2.5)*	-
Unknown	8 *(8.0)*	2 *(2.5)*	6 *(27.3)*
**PS**			
0	41 *(41.0)*	26 *(33.3)*	15 *(68.2)*
1	45 *(45.0)*	40 *(51.3)*	5 *(22.7)*
2	14 *(14.0)*	12 *(15.4)*	2 *(9.1)*
**ACE comorbidity Score**			
0 *(None)*	37 *(37.0)*	26 *(33.3)*	11 *(50.0)*
1 *(Mild)*	39 *(39.0)*	31 *(39.7)*	8 *(36.4)*
2 *(Moderate)*	17 *(17.0)*	16 *(20.5)*	1 *(4.5)*
3 *(Severe)*	7 *(7.0)*	5 *(6.5)*	2 *(9.1)*

Numbers in italics indicate percentage of total responses

Where ACE comorbidity scores were unavailable they were calculated by the researcher from the medical notes

ACE- Adult Comorbidity Evaluation, PS- Performance Status.* limited/extensive stage used for patients with small cell lung cancer

### Patient experience data

All patients either strongly agreed or agreed that they found the ePROMs service (MyChristie-MyHealth) easy to understand. Almost all (99%) felt ePROMs were easy to access and that the time taken to complete the questionnaires was appropriate. Finally, 97% reported that the timing of the text or email prompt to complete the questionnaires was appropriate.

When investigating the perceived impact of ePROMs on clinical care, 82% stated that using ePROMs improved communication with their oncology team and 88% agreed or strongly agreed that using ePROMs made them feel more involved in their care. Eighty-one participants felt that using ePROMs prompted them to seek medical advice sooner.

Eighteen patients reported receiving self-care advice through the MyChristie-MyHealth portal of which 14 said they found this advice helpful.

An evaluation of the of the free-text comment boxes found that the patients considered the questionnaire to be helpful, easy to use and a good method to aid communication with their clinical team. Some patients highlighted that they thought it was important that clinicians mentioned and demonstrated that they used the ePROMs responses during the clinical consultations. Other patients reported that the questions included were too rigid and suggested the inclusion of a free-text box at the end of the ePROM questionnaire so that they could add other comments on their health (Table 2).

**Table 2:** Patient reported experience measure (PREM) questionnaire responses

PREM questionnaire item	Response		
	All N = 100 n*(%)*	Lung N = 78 n*(%)*	Head and Neck N = 22 n*(%)*
I found the steps to get to MyChristie-MyHealth easy to follow			
Strongly agree	58 *(58.0)*	44 *(56.4)*	14 *(63.6)*
Agree	41 *(41.0)*	33 *(42.3)*	8 *(36.4)*
Disagree	1 *(1.0)*	1 *(1.3)*	0 *(0)*
Strongly disagree	0 *(0)*	0 *(0)*	0 *(0)*
I found MyChristie-MyHealth easy to understand			
Strongly agree	58 *(58.0)*	43 *(55.1)*	15 *(68.2)*
Agree	42 *(42.0)*	35 *(44.9)*	7 *(31.8)*
Disagree	0 *(0)*	0 *(0)*	0 *(0)*
Strongly disagree	0 *(0)*	0 *(0)*	0 *(0)*
The time it took me to fill out MyChristie-MyHealth was appropriate			
Strongly agree	55 *(55.0)*	40 *(51.3)*	15 *(68.2)*
Agree	44 *(44.0)*	37 *(47.4)*	7 *(31.8)*
Disagree	1 *(1.0)*	1 *(1.3)*	0 *(0)*
Strongly disagree	0 *(0)*	0 *(0)*	0 *(0)*
The time of day I received the text message from MyChristie-MyHealth was appropriate			
Strongly agree	48 *(48.0)*	36 *(46.1)*	12 *(54.5)*
Agree	49 *(49.0)*	40 *(51.3)*	9 *(40.9)*
Disagree	1 *(1.0)*	2 *(2.6)*	1 *(4.6)*
Strongly disagree	0 *(0)*	0 *(0)*	0 *(0)*
Using MyChristie-MyHealth improved my communication with my Christie team			
Strongly agree	38 *(38.0)*	29 *(37.2)*	9 *(40.9)*
Agree	44 *(44.0)*	33 *(42.3)*	11 *(50)*
Disagree	16 *(16.0)*	14 *(17.9)*	2 *(9.1)*
Strongly disagree	2 *(2.0)*	2 *(2.6)*	0 *(0)*
Using MyChristie-MyHealth made me feel more involved in my care			
Strongly agree	37 *(37.0)*	28 *(35.9)*	9 *(40.9)*
Agree	51 *(51.0)*	41 *(52.5)*	10 *(45.5)*
Disagree	11 *(11.0)*	8 *(10.3)*	3 *(13.6)*
Strongly disagree	1 *(1.0)*	1 *(1.3)*	0 *(0)*
I would like to fill in MyChristie-MyHealth			
More often	1 *(1.0)*	1 *(1.3)*	0 *(0)*
About as often as now	95 *(95.0)*	73 *(93.6)*	22 *(100)*
Less often	4 *(4.0)*	4 *(5.1)*	0 *(0)*
Using MyChristie-MyHealth prompted me to seek medical advice earlier when appropriate. (N/A responses excluded)			
	N = 32 n(%)	N = 27 n(%)	N = 5 n***(%)***
Strongly agree	12 *(37.5)*	10 *(37.0)*	2 *(40.0)*
Agree	14 *(43.7)*	13 *(48.2)*	1 *(20.0)*
Disagree	6 *(18.8)*	4 *(14.8)*	2 *(40.0)*
Strongly disagree	0 *(0)*	0 *(0)*	0 *(0)*
If you received advice through MyChristie-MyHealth to seek urgent medical help did you do so? (N/A responses excluded)			
	N = 26 n(%)	N = 23 n(%)	N = 3 n***(%)***
Yes	7 *(26.9)*	6 *(26.1)*	1 *(33.3)*
No	19 *(73.1)*	17 *(73.9)*	2 *(66.7)*
If you received self-care advice through MyChristie-MyHealth did you find it helpful? (N/A responses excluded)			
	N = 18 n(%)	N = 13 n(%)	N = 5 n***(%)***
Yes	14 *(77.8)*	10 *(76.9)*	4 *(80.0)*
No	4 *(22.2)*	3 *(23.1)*	1 *(20.0)*

Numbers in italics indicate percentage of total responses

### Clinician experience

#### Study population

Between June 2019 and July 2019, 11 oncologists specializing in lung and head and neck cancer completed the clinician experience questionnaire. Due to the set-up of the online platform, demographic data could not be collected. One questionnaire was returned with incomplete data (one question unanswered) but was felt to be sufficiently completed to be included in the analysis.

#### Clinician experience data

Eight clinicians (72.2%) reported that using ePROMs supported communication with their patients and six noted their use made consultations more patient-focused. Seven clinicians (63.6%) felt that ePROMs use had led to patients being more engaged during their consultations and 5 (45.5%) believed that patients using ePROMs were more engaged with their care as a whole.

Five clinicians (45.5%) felt that using ePROMs had contributed to their clinical decision making. Only one clinician reported that using ePROMs shortened their consultation time.

Some clinicians commented that whilst they thought the inclusion of ePROMs into clinical care was useful, integration into the electronic patient record would be a valuable step in ensuring that ePROMs were easier to use. Clinicians also commented that due to the lack of integration into the electronic patient record accessing and reviewing ePROMs was time consuming and frequently forgotten (Table 3).

**Table 3:** Clinician experience questionnaire responses

Clinician experience questionnaire item	Response			
	Strongly agree	Agree	Disagree	Strongly disagree
DrDoctor patient responses have influenced my clinical decision making N = 11	2 *(18.2)*	3 *(27.3)*	4 *(36.3)*	2 *(18.2)*
DrDoctor supported communication with my patients N = 11	2 *(18.2)*	6 *(54.5)*	1 *(9.1)*	2 *(18.2)*
DrDoctor has led to patients being more engaged with their care N = 11	2 *(18.2)*	3 *(27.3)*	6 *(54.5)*	0 *(0)*
DrDoctor supported patients to be more engaged during the consultation N = 11	3 *(27.3)*	4 *(36.3)*	3 *(27.3)*	1 *(9.1)*
DrDoctor has made consultations more focused on patient concerns N = 10 (1 missing data point)	2 *(20.0)*	4 *(40.0)*	3 *(30.0)*	1 *(10.0)*
DrDoctor has made consultations shorter N = 11	1 *(9.1)*	0 *(0)*	8 *(72.7)*	2 *(18.2)*

Numbers in italics indicate percentage of total responses

## Discussion

Historically, the use of PROMs in oncology care has been largely undertaken in the context of clinical research. Recently there has been a drive to incorporate regular ePROMs collection into routine cancer care [33, 34]. This study shows that the real-world collection of ePROMs as part of routine cancer care is acceptable to patients and clinicians and can have a positive impact on patient attitudes towards engagement with their care.

In this study, nearly all patients found ePROMs easy to use and understand which is similar to the findings from published literature on the use of PROMs in cancer care. Studies in a range of cancer sites and also in a palliative care setting have found that between 78.2% and 100% of patients found ePROMs easy to use [13, 35–41] and 97%-100% found them easy to understand [36–38]. It is worth noting that these studies have all used different electronic platforms to the current study but these findings support the idea that routine collection of ePROMs is acceptable to patients.

Our study found that 95% of patients surveyed were happy to continue completing ePROMs at every clinic visit which is higher than in previously published studies. In a study by Boyes et al., 75% of patients wished to complete a PROM questionnaire at each clinic visit [13] whilst only 60% of those in a study by Kallen et al. wanted to continue using ePROMs regularly as part of their clinical care [36]. The higher willingness to complete regular ePROMs in our evaluation may reflect the fact that only patients who had filled in at least one ePROMs questionnaire, and therefore more likely to continue to be compliant, were approached in this study, potentially introducing bias to the results. Furthermore, in the current evaluation the ePROMs initiative had been running for less than a year meaning patients may be less likely to have experienced questionnaire fatigue than in longer running studies. Another important finding is that over 80% of patients reported that completing the ePROMs questionnaires helped them to feel more involved in their care. Previous studies by Basch et al. demonstrated that 60–77% of patients felt more in control of their cancer care as a result of using ePROMs [40, 42]. It is possible that the different wording of the question in this study, using ‘involved’ rather than ‘in control’, may have led to the slightly increased agreement with this statement as patients have been found to experience a ‘lack of control’ whilst undergoing their treatment [40]. One limitation of this study is that it did not specifically investigate the barriers related to the routine collection of PROMs using an electronic platform.

Current literature is mixed when looking at the impact of the use of PROMs use on patient-clinician communication. Eighty-two percent of patients in our study felt that using ePROMs improved communication with their clinical team which is similar to a number of studies which have shown that between 51 and 95% of participants felt that the use of PROMs supported communication with their clinical team [36, 40, 42, 44, 45]. However, only 37% of respondents in a study by McLachlan et al. reported that PROMs improved communication with their clinical team [46] and Rosenbloom et al. did not find any statistically significant changes in patient satisfaction regarding communication when using PROMs as part of clinical care [47]. Reasons for this difference may be that the patients in the study by McLaughlin et al. were not undergoing treatment and only a small proportion were found to have high cancer needs which may have limited the effect. Furthermore, baseline satisfaction with communication was high prior to the implementation of PROMs in the Rosenbloom et al. study, which may have led to an element of ceiling effect.

Existing literature on PROMs echoes the comments made by patients in this study. The use of PROMs has been shown to help reassure patients [48] and better focus their thoughts on health related issues and symptoms during consultations [37, 44, 49, 50]. Patients in this study commented that clinicians were not systematically discussing their ePROMs questionnaire responses during consultations which has been found to be an issue in other studies. Boyes et al. found that only 3 of the 40 patients in their study recalled clinicians specifically mentioning PROMs responses during their consultations [13]. An important aim of ePROMs service improvement is therefore to raise the awareness of the importance of clinician’s review of the questionnaire and feedback to patients.

Most clinicians in this study reported that the use of ePROMs supported communication with patients (8/11) whilst just over half (6/10) reported that they led to consults being more patient focused. Interestingly although seven clinicians reported that the use of ePROMs led to patients being more involved in the consultation only five reported it improved engagement with their overall care. The current literature regarding the role of PROMs in supporting communication is very mixed [13, 51–54] and it appears that whilst 70%-100% of clinicians from a nursing or allied health care professional background feel that PROMs support communication [51, 52], only 50–67% of doctors agree with this statement [13, 53, 54].

To our knowledge, no previous literature has looked directly at the impact of ePROMs on making consultations more ‘patient-centred’. However 60% of clinicians in a study by Berry et al. found that the use of PROMs helped to guide consultations [53] and 67% in a study by Mark et al. reported that PROMs helped to focus consultations [37].

This study found that 45% of clinicians reported that patients’ ePROMs responses contributed to their decision-making. A study by Moore et al. [45] looking at using ePROMs as part of routine cancer care in haematological malignancies found similar results to this study in that just over 40% of clinicians reported taking action after looking at the results of ePROMs. However, an earlier study conducted at the Christie showed this percentage to be much higher (79.5%) [38]. It is important to note that in the earlier study, patient responses to the ePROMs questionnaires were available within the electronic patient record rather than on a separate platform as was the case in this study. This was reported by clinicians as a potential barrier to accessing ePROMs responses prior to the consultation and could contribute to the difference in the results. This issue has since been rectified. The ePROMs responses have been available in the electronic patient record for the clinical team to review since March 2020.

Approximately two-thirds of the patients in this study who stated they received advice to seek urgent medical help reported that they did not heed this advice. Reasons given by patients for not seeking urgent help were that the patient was due to see their oncology team in the very near future or that the symptom was long-standing and being managed. This has highlighted an important area for ongoing study to explore further patients’ reasons for not heeding the urgent medical advice prompts and whether there needs to be alterations in the threshold for the alerts.

One limitation of this study is that the clinician experience questionnaire was not completed by all clinicians involved in clinics using ePROMs, again potentially leading to bias in the results. Another area for potential bias was noted as all questions were phrased in a positive way and no negative phrasing was used. This was primarily to ensure they were easy to understand and to keep the number of questions as low as possible to avoid questionnaire fatigue but it is acknowledged that this could lead to more positive responses. In the same vein, although the PREM questionnaires were collected anonymously the patients were approached to complete the questionnaire by a member of the MyChristie-MyHealth team. This could therefore introduce potential bias as patients may not want to respond negatively about a service that is providing their cancer care.

Future directions for the project include gaining experience data from non-completers as well as the continued review of patient and clinician experience to aid future development of the MyChristie-MyHealth service. A further roll out of the initiative to all patient groups and the development of ad-hoc and responsive ePROMs service can help create an adaptive, patient-centred approach to routine cancer care (Table 4).

**Table 4:** Future directions for the MyChristie-MyHealth project

Area	Key learning points	Future directions	Action plan
Patient experience	Regular collection of ePROMs as part of routine cancer care is acceptable and feasible	Gain experience data from non-completers	Repeat experience study evaluating non-completers
	ePROMs make patients feel more involved in their care	Ongoing feedback from patients to develop service	ePROMs patient coordinator as point of contact in clinic
Clinician experience	ePROMs support communication and patient engagement	Further clinician experience data needed	Clinician experience review (aim 100% feedback)
	Integration into the electronic patient record (EPR) is essential	Explore reasons for non-engagement with ePROMs	
Developing MyChristie-MyHealth service to improve patient centered care	ePROMs help patients feel more involved in their care and consultations more patient focused	Roll out of ePROMS into all disease groups and clinics	
	Increased need of virtual follow-up during/since the COVID pandemic	Use of ePROMs for adaptive/virtual follow-up	
		Develop ‘ad-hoc’ ePROMs service with real-time clinical review	

## Conclusion

This study has shown that the use of regular ePROMs collection in routine cancer care is not only feasible and acceptable to patients and clinicians alike, but can also lead to improved communication between patients and their oncology teams. Furthermore, ePROMs can help to make patients feel more involved in their care and be more engaged in consultations. Our findings will help other centres who may be considering the implementation of ePROM into routine care and provide some ideas of further work that is required in this setting. Further research looking specifically at patients who did not complete the ePROMs, enhancing engagement of clinicians with the service and constant review and evaluation of the MyChristie-MyHealth initiative is needed moving forward to optimize the benefits to patients.

## Supplementary Information

**Additional file 1.** Examples of lung cancer ePROMs questions

**Additional file 2.** Examples of head and neck cancer ePROMs questions

## Data Availability

he datasets used in the current study are available on request from the corresponding author.

## Abbreviations

ACE: Adult comorbidity evaluation
ePROM: Electronic patient reported outcome measure
EQ-5D-5L: European quality of Life- 5 dimensions-5 levels
H&N: Head and neck
PREM: Patient reported experience measure
PROM(s): Patient reported outcome measure(s)
PS: Performance status
QoL: Quality of life
