# Supplementary figures and images for: Patient and clinician-reported experiences of using electronic patient reported outcome measures (ePROMs) as part of routine cancer care
Source: J Patient Rep Outcomes. 2023 May 4;7:42. doi: 10.1186/s41687-023-00544-4 (PMC10160312; doi:10.1186/s41687-023-00544-4)

# Appendix 1


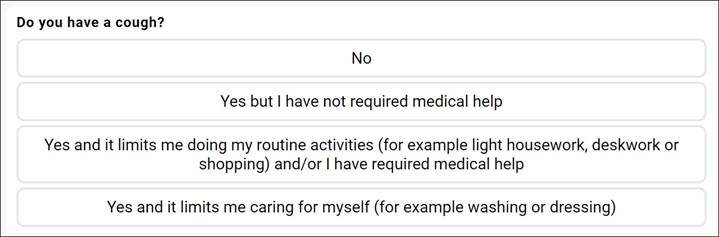


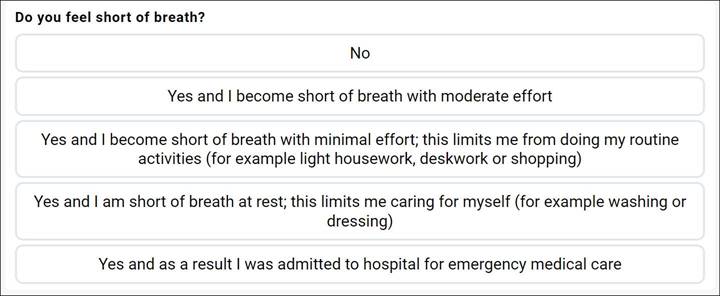

Supplement: Supplementary file 1 — Additional file 1. Examples of lung cancer ePROMs questions [file 41687_2023_544_MOESM1_ESM.docx]

# Appendix 2


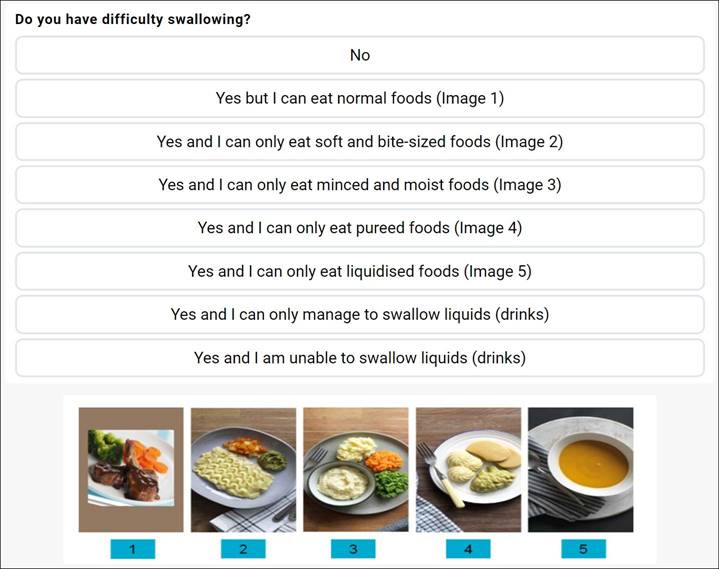


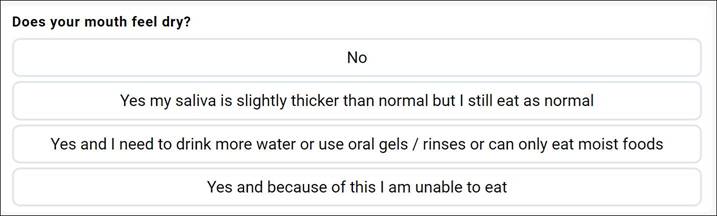

Supplement: Supplementary file 2 — Additional file 2. Examples of head and neck cancer ePROMs questions [file 41687_2023_544_MOESM2_ESM.docx]
